# Motor/Nonmotor Symptoms and Progression in Patients with Parkinson's Disease: Prevalence and Risks in a Longitudinal Study

**DOI:** 10.1155/2020/2735361

**Published:** 2020-06-20

**Authors:** Asako Yoritaka, Yasushi Shimo, Taku Hatano, Nobutaka Hattori

**Affiliations:** ^1^Department of Neurology, Juntendo University Koshigaya Hospital, Koshigayashi, Saitama 343-0032, Japan; ^2^Department of Neurology, Juntendo University School of Medicine, Tokyo 113-8421, Japan

## Abstract

We previously assessed the prevalence and risks of motor/nonmotor symptoms in a large sample of Japanese patients with Parkinson's disease. In the present study, we longitudinally assessed the prevalence and risk of motor/nonmotor symptoms, changes in treatment, disease progression, and death in patients with Parkinson's disease. We enrolled 1,227 patients diagnosed and treated at our hospital in Tokyo at first evaluation. We were able to follow-up 445 patients until the second evaluation, 7.4 years later. Using Kaplan–Meier survival curves and the Cox proportional-hazards model in 1,227 patients, motor/nonmotor symptoms were analyzed in association with the following events: pain, wearing-off, camptocormia, psychosis, orthostatic hypotension, pneumonia, tube feeding, modified Hoehn and Yahr stages (H–Y) 3 and 4 of the on state, and death. The mean age (standard deviation) at the first evaluation was 67.2 (9.9) years, while the mean ages at onset and disease duration were 57.8 (11.7) years and 9.3 (6.6) years, respectively. The mean H–Y of the on state was 2.7 (1.1) at the first evaluation. Age at onset and duration of levodopa use decreased the hazard ratios (HRs) (0.968 and 0.910, respectively) for wearing-off. Female sex increased the HRs (1.414) for wearing-off and decreased the HRs for orthostatic hypotension (0.540) and pneumonia (0.510). Older age at onset increased the HR for psychosis (1.035), orthostatic hypotension (1.033), H–Y 3 (1.048) and 4 (1.071), pneumonia (1.123), tube feeding (1.140), and death (1.095). Early onset of orthostatic hypotension itself increased the HR for numerous events, especially for death (0.893). Our results indicated that age, sex, and some nonmotor symptoms may predict many Parkinson's disease-related events. In addition, these data may provide a useful reference for the clinical course of Parkinson's disease.

## 1. Introduction

Dopamine replacement with levodopa or dopamine agonists (DAs) results in marked improvement of motor symptoms (MS), disability, and patient survival of Parkinson's disease (PD) [1, 2]. However, levodopa use is also associated with the development of motor complications, such as dyskinesia and wearing-off in patients with advanced PD, substantially contributing to overall disability and affecting quality of life. In addition to MS, various non-MS (NMS), such as pain, orthostatic hypotension, sleep disturbance, and psychosis, and the adverse effects of antiparkinsonian drugs limit the medication dose and the ability to prescribe other antiparkinsonian agents. In addition to long-term follow-up studies conducted after randomized clinical trials [3–5], some longitudinal studies have recently examined MS/NMS hazards in patients with PD [6–8]. In the present longitudinal study, we followed up patients for 7.4 years in continuation to our previous study [9, 10] and investigated the influence of sex, age at onset, onset symptoms, drug use, and preceding symptoms on the prevalence of MS/NMS. We focused on milestone symptoms affecting quality-of-life changes and disease progression in a large sample of Japanese patients with PD in a real-world setting. Therefore, we included patients with other organic diseases or patients after treatment for malignancy. Moreover, we added the preceding MS/NMS as factors in this analysis and examined the possibility that the symptoms would have predictive value.

## 2. Materials and Methods

### 2.1. Patients

We enrolled patients who had been diagnosed with PD at our neurology clinic at Juntendo Hospital in Tokyo, by a board-certified neurologist using the UK Brain Bank diagnostic criteria for PD [11], between January and June 2010, and retrospectively examined the prevalence and risk of MS/NMS at the “first evaluation [9, 10].” Patients with dementia with Lewy bodies and other forms of parkinsonism were excluded [9]. We followed up the patients until the “second evaluation” performed between March 2017 and February 2018. The present study was approved by the Juntendo Hospital Institutional Ethics Committee, and all patients provided written informed consent.

### 2.2. Evaluations

In continuation to our previous report, hospital charts at every visit during the follow-up period were reviewed by a single author (A. Y.) with regard to the following events: pain, wearing-off, camptocormia, psychosis, orthostatic hypotension, pneumonia, tube feeding, and modified Hoehn and Yahr stages (H–Y) 3 (including 2.5) and 4 of the on state, and death. “Onset,” “pain,” “camptocormia,” “orthostatic hypotension,” and “psychosis” were defined in a previous report [9]. Therefore, pain and the camptocormia associated with spinal diseases, bone fractures, and pain due to other diseases were not included in the events. Other NMS, such as depression and cognitive impairment, were not examined because their onset was unclear and the patients were not regularly examined using tools, such as the NMS questionnaire [12–14]. The daily levodopa equivalent dose (LED) was calculated as previously described [15]. The zonisamide and istradefylline LEDs were unknown and thus could not be included in the total LED.

### 2.3. Statistical Analyses

Statistical analyses were performed using SPSS (ver. 20.1; IBM Inc., Chicago, IL, USA). Data are expressed as the mean (standard deviation [SD]). Kaplan–Meier (K–M) time-to-event curves and log-rank tests were used to estimate the absolute risk of each event. The factors selected were as follows: sex, age at onset (early onset <60 years or late onset ≥60 years, divided by the approximate mean age at onset), onset symptoms (tremor or others), order of drugs (levodopa first or extra levodopa first (other antiparkinsonian drugs)), and presence or absence of other events (pain, wearing-off, psychosis, and orthostatic hypotension) before the focused event (for example, the focused event-pain, occurred in 2016, and the other event-psychosis, occurred in 2012 and was regarded as “presence of other event,” occurred in 2017, and was regarded as “absence of other event”). Cox proportional-hazards modeling was used to calculate hazard ratios (HRs) and 95% confidence intervals (CIs) for differences among the following variables: sex, age at onset, onset symptoms (tremor or others), duration until the start of drug use (levodopa, or antiparkinsonian drugs except levodopa), daily levodopa dose and total LED at first evaluation, and duration to the other events from disease onset (pain, wearing-off, psychosis, and orthostatic hypotension). Subgroup analysis was performed based on sex and age at onset. Proportional hazards were assessed using graph log-log plots. Statistical tests were two-sided, and the level of significance was set to *p* < 0.05.

## 3. Results

**Figure 1:**
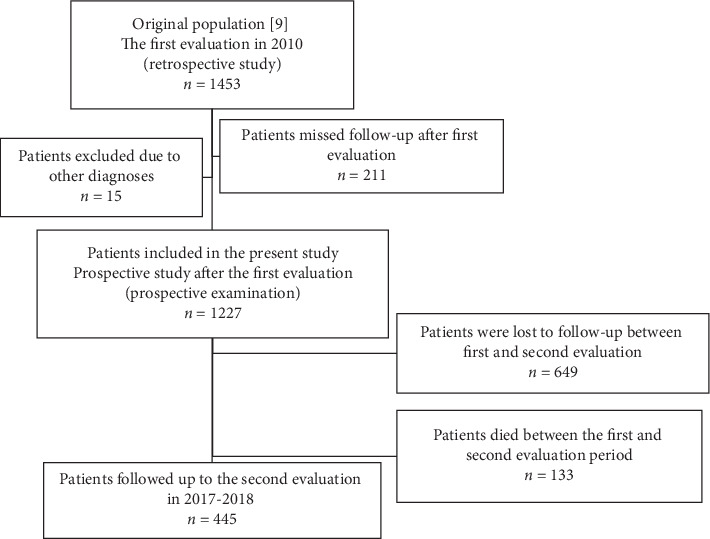
Flow chart of patients included in the analysis.

Although we enrolled 1,453 patients with PD at the first evaluation, we excluded patients who did not return during the follow-up period (Figure 1) and 15 patients who received a different diagnosis (e.g., nine patients were diagnosed with progressive supranuclear palsy [16]); this longitudinal study included data from 1,227 patients (551 men).

All 445 patients were followed up until the second evaluation; some patients were lost to follow-up due to progression of symptoms, change in clinic, or unknown causes. We defined “the last evaluation” as the last visit of all patients during follow-up.

**Figure 2:**
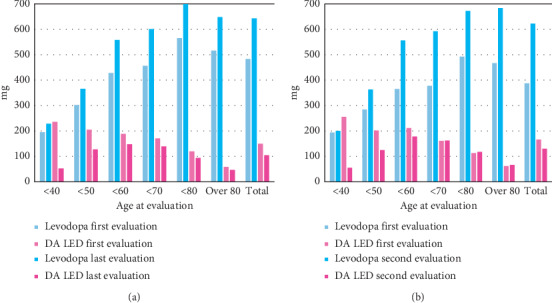
Daily levodopa dose and levodopa equivalent dose for DA based on age at each evaluation. (a) Daily dose of all 1227 patients included in this study, provided that at the last evaluation, the period of follow-up varied. (b) Daily dose of 445 patients who were followed up until the second evaluation. DA: dopamine agonists, LED: levodopa equivalent dose.

Clinical findings and medication data for the included patients are shown in Table 1 and Figure 2. The mean age (SD) of the 1227 patients at the first evaluation was 67.2 (9.9) years, the mean age at onset was 57.8 (11.7) years, and the mean disease duration was 9.3 (6.6) years. The duration from disease onset to the first clinical day at our hospital, duration from the first clinical day to the last evaluation at our hospital, and duration of treatment at all hospitals were 3.4 (6.9), 10.1 (5.7), and 11.7 (6.9) years, respectively. 47.7% of 1227 patients had tremor as the onset symptom. The percentages of patients with PD who also had hypertension, diabetes mellitus, dyslipidemia, cerebrovascular disease, and malignant tumors were 17.8%, 12.0%, 5.4%, 5.9%, and 6.0%, respectively. Mean H–Ys were 2.7 (1.1) at the first visit and 3.5 (1.1) at the last evaluation. Apomorphine injection was administered in 18 cases, and deep brain stimulation (DBS) in 76 cases. Total LED increased (*p* < 0.001) between the evaluations, and the LED of DA decreased (*p* < 0.001) (Table 1) in 445 patients.

**Figure 3:**
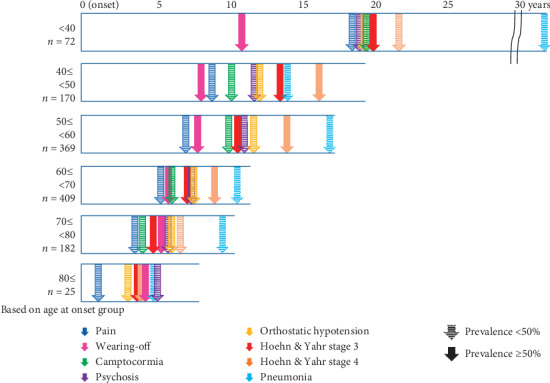
Duration from disease onset to each event based on age at onset. Squares indicate the duration from onset to death, while arrows indicate the mean duration from onset to each event.

The prevalence and mean duration from PD onset to the examined events are shown in the first column of Table 2.  Figure 3 shows the duration from disease onset to the onset of each event based on the age at onset. The mean duration from disease onset to event onset was longer in patients who had disease onset at a younger age; and those over the age of 80 years at disease onset experienced various events approximately 5 years after onset.

**Figure 4:**
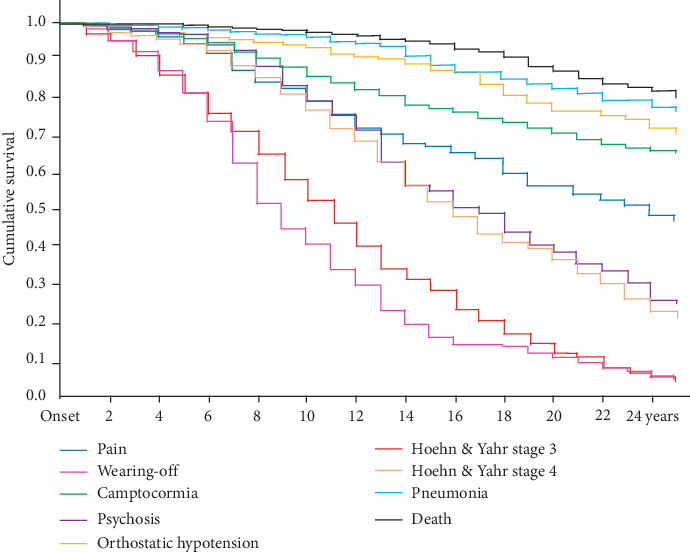
Kaplan–Meier time-to-event curves for motor and nonmotor symptoms and death.

Survival rates obtained from K-M curves for all events following PD onset are shown in Table 2 and Figure 4. We show the significant influencing factors for each event in Table 2. Cox HRs are shown in Tables 3–5 (subgroup analysis).

### 3.1. Pain

The duration from disease onset to pain rates of 25% and 50% was 10.8 (0.5) years and 23.2 (1.5) years, respectively. Log-rank tests revealed that female sex (*p* = 0.005) and older age at onset (*p* < 0.001) increased the pain rate. Early experience of wearing-off, psychosis, and orthostatic hypotension along with an early start of antiparkinsonian drugs except levodopa, increased the HRs for pain.

### 3.2. Wearing-Off

The duration from disease onset to wearing-off rates of 25%, 50%, and 75% was 5.7 (0.2) years, 8.4 (0.2) years, and 13.5 (0.4) years, respectively. Female sex and symptoms other than tremor at onset (*p* < 0.001) significantly increased the wearing-off rate. While female sex, younger age at onset, symptoms other than tremor at onset, early start of levodopa, and early experience of pain and orthostatic hypotension increased the HRs for wearing-off.

Dyskinesia was a prevalent motor fluctuation, with 36.9% at the first evaluation and 44.3% at the last evaluation; additionally, 94% of the patients with dyskinesia had wearing off.

### 3.3. Camptocormia

The duration from disease onset to a camptocormia rate of 25% was 16.3 (1.3) years. Female sex (*p*=0.033) and older age at onset (*p*=0.048) significantly increased the camptocormia rate. Early experience of pain increased the HRs for camptocormia.

### 3.4. Psychosis

The duration from disease onset to psychosis rates of 25%, 50%, and 75% was 10.8 (0.3) years, 16.1 (0.5) years, and 25.4 (1.7) years, respectively. Older age at onset (*p* < 0.001) significantly increased the psychosis rate. Older age at onset, early experience of pain, wearing-off, and orthostatic hypotension also increased the HRs for psychosis.

### 3.5. Orthostatic Hypotension

The duration from disease onset to an orthostatic hypotension rate of 25% was 23.4 (1.5) years. Male sex (*p*=0.006), older age at onset (*p* < 0.001), and use of levodopa as first treatment (*p*=0.030) significantly increased the orthostatic hypotension rate. Male sex, early experience of wearing-off and psychosis, and early start of levodopa increased the HRs for orthostatic hypotension.

### 3.6. H–Y 3 and 4

The duration from disease onset to an H–Y 3 rate of 25%, 50%, and 75% was 6.1 (0.2) years, 10.5 (0.2) years, and 15.8 (0.4) years, respectively. The duration from disease onset to an H–Y 4 rate of 25%, 50%, and 75% was 10.1 (0.3) years, 15.7 (0.4) years, and 23.3 (0.7) years, respectively. Female sex (*p*=0.016), absence of psychosis (*p* < 0.0001), and levodopa first significantly increased the H–Y 3 rate. Older age at onset (*p* < 0.0001) and absence of wearing-off (*p* < 0.0001) significantly increased the rates of H–Y 3 and 4. Older age at onset, along with symptoms other than tremor at onset and early experience of wearing-off, psychosis, and orthostatic hypotension, increased the HRs for both H–Y 3 and 4. Early experience of pain increased the HRs for H–Y 3.

### 3.7. Pneumonia

Male sex (*p*=0.003), older age at onset (*p* < 0.001), absence of wearing-off (*p*=0.011), and use of levodopa as first treatment (*p*=0.034) increased the pneumonia rate. Male sex, older age at onset, and early experience of orthostatic hypotension increased the HRs for pneumonia.

### 3.8. Tube Feeding

Older age at onset (*p* < 0.001), absence of wearing-off (*p*=0.027), and use of levodopa as first treatment (*p*=0.048) increased the tube feeding rate. Older age at onset and early experience of orthostatic hypotension increased the HRs for tube feeding.

### 3.9. Death

A total of 133 patients (70 women) died between the first and second evaluations due to PD-related causes, including aspiration pneumonia due to disease progression (*n* = 63), malignant tumors (*n* = 11), other diseases (*n* = 32), or unknown causes (*n* = 27). The mean age among these patients was 74.5 (8.1) years, while age at onset was 61.0 (11.9) years. The mean H–Y at the first evaluation was 3.1 (1.1), while the mean H–Y at the last examination was 4.1 (0.8). The mean body mass index was 19.3 kg/m^2^ (normal range: 18.5–25.0 (BMI JAPN) [17]), and it was not lower than that of the other patients (data not shown). The duration between psychosis onset and death was 5.2 (4.0) years, while the duration between pneumonia onset and death was 1.3 (1.9) years. Older age at onset (*p* < 0.001), absence of pain (*p*=0.039), absence of wearing-off (*p*=0.003), and presence of psychosis (*p*=0.041) decreased cumulative survival. Older age at onset, early experience of pain, and orthostatic hypotension increased the HRs for death.

## 4. Discussion

In this study, we assessed MS/NMS, disease progression, and treatment among Japanese patients with PD. While the ratio of women to men was higher in our study, as in a previous Japanese study, our results indicated that age at PD onset was higher in women than in men, in accordance with previous findings [18, 19]. Female patients were at risk of experiencing wearing-off and reaching H–Y 3 and 4, while male patients were at risk of orthostatic hypotension and pneumonia. Early-onset PD was a risk factor for wearing-off. Older age at onset was a risk factor for orthostatic hypotension, psychosis, H–Y 3 and 4, pneumonia, tube feeding, and death. Symptoms other than tremor at onset were a risk factor for wearing-off and H–Y 3 and 4. Female patients who experienced pain were at a risk of camptocormia, psychosis, and death.

Previous studies have reported that motor fluctuations occur in 50% or more of patients with PD treated for longer than 5 years [20, 21]. In one previous study, the rate of wearing-off was 21.3% in the 5th year, 59.4% in the 10th year, and 73.2% in the 15th year [18], similar to the present findings. Likewise, in our study, female sex and earlier onset were risk factors for wearing-off [20], which may have been due to lower body weight among women. In this study, the daily doses at first evaluation were significantly different (*p* < 0.01) between men (8.6 mg/kg) and women (10.5 mg/kg). In addition, the daily doses were significantly different (*p* < 0.001) between the group with wearing-off (11.7 mg/kg) and the group without wearing-off (7.0 mg/kg). However, no differences were observed in the body mass index. Regarding dyskinesia, the daily levodopa dose/kg in the patients with dyskinesia was significantly higher (*p* < 0.001) than in those without dyskinesia. Higher levodopa bioavailability in women [22] might explain the higher rate of motor complication. In our study, earlier initiation of levodopa treatment increased the risk of wearing-off, whereas the order in which drugs were prescribed (levodopa or other drugs) did not affect wearing-off. Clinical trials studies [3–6] reported no differences in the onset of wearing-off over 10 years of follow-up. The nominal (actual) dose of levodopa at the onset of wearing-off was associated with the HRs [23], although levodopa dose at the first evaluation and the cumulative dose of levodopa until the onset of wearing-off were not a risk factor in our study [10]. As in the Italian and Ghanaian studies, longer disease duration rather than the duration of levodopa therapy exposure was associated with wearing-off [24].

Previously, we reported that there was an association between nonergot DA and camptocormia [9], leading us to decrease or discontinue suspicious nonergot DA administration at earlier states in many patients in this study. In this study, pramipexole treatment was discontinued in 54% of patients due to not only camptocormia, but also increased psychosis and sleepiness, or dopamine dysregulation syndrome. The LED of DA was decreased from 19.8% of the total LED to 11.9% from the first to the second evaluation.

A pathology-based study revealed that hallucinations are one of the developmental milestones leading to death [25]. Conversely, a previous study reported that hallucinations occur more frequently among patients with early PD treated with DA than among those treated with placebo or levodopa [26]. Mini Mental State Examination (MMSE) scores decreased from 22.0 (9.3) (*n* = 167) at the first evaluation to 15.7 (11.7) at the second evaluation of patients with psychosis in this study. In contrast, MMSE scores decreased from 25.6 (7.5) (*n* = 118) to 22.2 (11.5) among patients without psychosis. Although this difference was not significant, this suggests that patients with psychosis exhibit more rapid cognitive decline than patients without psychosis. In our previous first evaluation study [9], HRs of psychosis were not significant in the cumulative dose of total dopamine agonist and cumulative dose of trihexyphenidyl, amantadine, or selegiline until the onset of psychosis; and early start of antiparkinsonian drugs, except for levodopa, did not induce psychosis; therefore, we assumed that these drugs did not increase psychosis rate. There were no differences in the HRs between patients treated with levodopa first and those treated with other drugs first; in our study, although many patients had to discontinue or decrease the doses of drugs other than levodopa. NMS might lead to use of levodopa as the main treatment.

Autonomic dysfunction is associated with an increased risk of more rapid progression in patients with PD [27, 28]. In our study, orthostatic hypotension, as a factor, had greater influence than did the other analyzed symptoms or timing of drugs on the analyzed events, such as disease progression or death, and these results might be linked to the network deterioration in cortical regions in patients with autonomic dysfunction [29]. The lack of tremor symptoms at onset predicted poor prognosis in a Cox proportional-hazards analysis [2, 30]. NMS profile and severity varied according to the motor phenotype. In the PD population, patients with a postural instability gait disorder phenotype who have more axial involvement associated with advanced disease and poor motor response have a higher risk of higher NMS burden [31]. Male sex, especially in elderly patients, was a primary risk factor for orthostatic hypotension. The rate of male patients without tremor symptom at onset was higher than that of female patients (*p* < 0.05).

In our study, disease progression to H–Y 3 was slower than that reported by a previous Japanese study (30.2%, 57.2%, and 83.5% by the end of the 5th, 10th, and 15th years, respectively) [18]. In accordance with previous findings [18], female sex was among the risk factors for progression to H–Y 3 in this study. The progression to H–Y 3 and 4 was slower in men than in women, possibly because of the postural instability being dominant in women [32]. The previous study reported death rates of 0.7%, 10.2%, and 18.7% by the end of the 5th, 10th, and 15th years [18], which were higher than in our study.

We believe that the patients received the best treatments available at that time, as the H–Y improved for some period in many patients over the course of the study. In Japan, nonergot DAs, pramipexole, immediate- and extended-release ropinirole, and rotigotine were launched between 2004 and 2013. Coverage for DBS also began in 2000, while the nondopaminergic parkinsonian drugs entacapone, zonisamide, and istradefylline became available between 2009 and 2013. Long-term care insurance started in 2000 and supported daily rehabilitation of patients in their homes or neighborhood facilities and monitoring of medication compliance. This improved insurance system or increase in treatment options may explain the relative improvements observed in this study.

This study possesses some limitations of note. Although we conducted a longitudinal analysis, retrospective data were included in the first evaluation and were mixed with the prospective data of the second evaluation. We included lack of follow-up data for numerous patients, many of whom were unable to attend follow-up visits due to progression of symptoms or change of the local treating physician. We did not remove the confounding factors from this real-world setting study, which was not controlled. We included neither the clinical scale of MS (i.e., unified Parkinson's disease rating scale) nor the scale of non-MS/signs such as cognition, mood disorders, and rapid eye movement sleep behavior disorders. The causes of death were determined based on clinical rather than pathological findings. In addition, we did not utilize questionnaires to assess presence of some symptoms, so the prevalence rates for some events may have decreased. Our population involved uncontrolled heterogenous patients of Japanese ethnicity, who came to our hospital even from distant areas (the hospital is conveniently located to be reached from various areas).

## 5. Conclusions

In conclusion, we investigated the duration and prevalence of various symptoms, complications, and death in a large cohort of patients with PD in a real-world setting. Our results indicated that age and sex and certain symptoms may predict many PD-related events.

## Figures and Tables

**Table 1:** Clinical findings and prescribed drugs of the enrolled 1227 patients with Parkinson's disease and the 445 patients followed up through the second evaluation.

		All cases		Follow-up to the second evaluation		
		*n* = 1227		*n* = 445		
		First evaluation	Last evaluation	First evaluation	Second evaluation	Paired *t*-test *p* (1st and 2nd)
Follow-up	years	4.4 ± 2.9		7.4 ± 1.2		
Age at onset	years	57.8 ± 11.7		54.9 ± 12.3		
Age	years	67.2 ± 9.9	71.5 ± 9.5	62.5 ± 10.7	69.8 ± 10.5	
Sex (female)		676	55%	249	56%	
Hoehn and Yahr stage		2.7 ± 1.1	3.5 ± 1.1	2.30 ± 1.1	3.3 ± 1.1	<0.001
Total LED	mg	756 ± 423.0	873.6 ± 384.3	697.8 ± 433.0	896.5 ± 367.2	<0.001
Dopamine agonist LED	mg	149.5 ± 140.9	104.3 ± 138.0	189.7 ± 153.0	131.9 ± 146.1	<0.001
Levodopa	*n*	1088	1202	356	434	
	mg	541.9 ± 303.7	655.9 ± 287.1	479.3 ± 288.3	636.6 ± 259.2	<0.001
Pramipexole	*n*	777	312	299	92	
	mg	2.0 ± 1.3	2.3 ± 1.1	2.0 ± 1.3	2.3 ± 1.1	
Ropinirole	*n*	184	122	80	68	
	mg	7.3 ± 3.2	8.8 ± 2.9	8.0 ± 3.7	10.0 ± 3.8	
Rotigotine	*n*	—	149	—	97	
	mg	—	8.1 ± 4.0	—	8.3 ± 4.1	
Pergolide	*n*	325	39	108	8	
	mg	1.0 ± 0.5	1.0 ± 0.2	1.0 ± 0.5	1.3 ± 0.2	
Cabergoline	*n*	329	12	117	0	
	mg	2.4 ± 1.3	2.6 ± 0.3	2.7 ± 1.4	0	
Selegiline	*n*	517	228	178	103	
	mg	4.9 ± 2.8	5.1 ± 2.2	5.1 ± 2.9	5.2 ± 2.4	
Entacapone	*n*	264	372	100	192	
	mg	486.2 ± 229.2	533.6 ± 285.1	502.1 ± 240.0	535.2 ± 319.0	
Istradefylline	*n*	—	75	—	50	
	mg	—	29.9 ± 7.6	—	29.6 ± 9.9	
Trihexyphenidyl	*n*	480	169	168	42	
	mg	3.2 ± 1.9	2.7 ± 1.0	3.6 ± 2.1	3.3 ± 1.0	
Amantadine	*n*	320	106	150	110	
	mg	166.7 ± 91.1	176.0 ± 85.3	181.7 ± 94.4	194.3 ± 91.8	
Zonisamide	*n*	106	291	46	151	
	mg	10.9 ± 25.0	44.4 ± 24.9	45.4 ± 17.5	47.3 ± 30.0	
Droxidopa	*n*	105	79	27	30	
	mg	382.9 ± 118.9	498.1 ± 134.6	396.4 ± 107.3	464.5 ± 127.9	
Cholinesterase inhibitor	*n*	89	259	13	87	
Memantine	*n*	—	89	—	40	
Midodrine	*n*	44	66	11	23	

**Table 2:** Kaplan–Meier survival of events in the patients with Parkinson's disease. The factors selected were as follows: age at onset (early onset <60 years or late onset ≥60 years), sex, order of drugs (levodopa first or extra levodopa first (other antiparkinsonian drugs)), and presence or absence of other events before the event (pain, wearing-off, orthostatic hypotension, and psychosis). The categories shown in the table affected the incidence rate of factors.

Events	Prevalence of events, mean duration from disease onset years (SD)		Disease duration and Kaplan–Meier proportional curve (%)											
		Factor	2nd year	4th year	6th year	8th year	10th year	12th year	14th year	16th year	18th year	20th year	25th year	Log-rank test
		*n*	1222	1192	1119	1015	840	660	480	355	253	183	76	*p*
Pain	31.0% 7.3 (5.5) years	**Total**	**2.4**	**6.0**	**10.7**	**16.1**	**22.4**	**28.4**	**32.1**	**35.2**	**40.4**	**42.5**	**53.5**	
		Male	2.8	4.8	9.0	13.3	18.1	24.8	28.8	31.8	36.9	41.2	—	0.005
		Female	2.8	7.1	12.1	18.6	26.1	31.4	34.7	38.4	43.3	46.5	58.3	
		<60.0 years old	1.2	4.0	8.3	13.3	19.1	24.9	27.2	29.7	34.3	39.6	49.7	<0.0001
		≥60.0 years old	3.8	7.9	13.3	19.3	26.0	34.0	40.0	46.4	53.7	60.3	—	

Wearing-off	72.0% 7.7 (4.5) years	**Total**	**2.6**	**13.1**	**27.4**	**47.2**	**59.8**	**70.2**	**78.1**	**83.6**	**85.7**	**88.6**	**94.3**	
		Male	2.5	12.8	24.5	43.4	52.5	63.9	72.9	81.4	83.1	85.7	91.4	<0.0001
		Female	3.1	13.4	29.8	50.4	65.7	75.2	82.2	85.7	88.4	91.5	96.8	
		Tremor onset	1.8	10.6	23.7	40.8	51.6	62.9	72.2	77.6	80.8	85.5	93.6	<0.0001
		Other onset	3.1	15.0	30.0	52.4	66.6	76.4	82.4	88.5	89.7	90.8	95.6	

Camptocormia	22.7% 8.8 (5.7) years	**Total**	**2.5**	**5.1**	**9.1**	**11.9**	**16.8**	**17.5**	**21.1**	**24.3**	**26.7**	**29.3**	**33.8**	
		Male	2.7	3.4	6.5	9.7	13.1	13.1	18.1	22.1	23.7	24.7	26.5	0.033
		Female	3.1	6.4	9.7	13.8	16.8	19.8	23.5	26.8	29.8	32.8	36.7	
		<60.0 years old	2.9	4.0	6.3	9.2	11.6	14.9	19.0	22.3	25.0	27.8	31.1	0.048
		≥60.0 years old	11.9	16.1	10.2	14.5	18.2	20.3	23.3	26.7	26.7	—	—	

Psychosis	42.5% 9.6 (6.0) years	**Total**	**2.0**	**3.7**	**7.4**	**12.6**	**10.8**	**29.7**	**42.3**	**49.7**	**56.6**	**61.6**	**74.8**	
		<60.0 years old	1.9	2.7	4.7	8.2	12.3	17.3	29.0	36.6	43.8	50.1	66.7	<0.0001
		≥60.0 years old	2.2	4.7	10.3	16.6	30.9	46.1	62.2	72.6	80.4	100.0	—	

Orthostatic hypotension	12.1% 10.0 (3.8) years	**Total**	**0.6**	**1.6**	**2.9**	**5.2**	**6.4**	**9.5**	**12.8**	**14.2**	**18.1**	**21.6**	**29.2**	
		Male	0.4	1.5	3.3	6.1	8.1	11.8	16.6	18.1	20.8	27.6	33.8	0.006
		Female	0.8	1.5	2.5	4.4	5.0	7.7	9.6	10.4	15.1	17.6	23.2	
		<60.0 years old	0.0	0.5	1.2	2.8	4.0	6.1	8.0	9.8	13.2	16.6	23.9	<0.0001
		≥60.0 years old	1.0	2.7	4.5	7.5	9.3	13.7	20.7	20.7	26.5	31.4	—	
		Extra levodopa first	0.3	1.2	2.1	3.8	4.5	7.5	11.5	12.6	15.0	18.4	24.9	0.030
		Levodopa first	0.9	2.0	4.1	7.2	9.3	12.6	14.4	16.1	22.1	25.3	32.5	

Hoehn and Yahr stage 3	78.1% 9.7 (6.9) years	**Total**	**4.9**	**13.4**	**23.9**	**35.0**	**46.9**	**58.4**	**68.4**	**75.7**	**81.8**	**87.5**	**93.4**	
		Male	3.8	12.1	21.8	32.4	43.9	55.0	66.2	72.9	78.7	85.5	92.4	0.016
		Female	5.7	14.4	25.6	37.2	49.3	61.3	70.2	78.0	86.2	89.0	94.2	
		<60.0 years old	1.5	5.1	10.4	16.5	24.9	36.3	48.8	58.8	68.4	77.7	88.3	<0.0001
		≥60.0 years old	8.2	21.6	37.4	53.7	69.6	82.0	90.0	95.3	100.0			
		Wearing-off (−)	9.9	26.5	42.9	56.0	68.4	77.1	82.3	86.6	90.0	94.6	96.6	<0.0001
		Wearing-off (+)	0.3	2.2	7.8	17.5	29.3	43.4	57.1	67.0	75.1	82.1	90.8	
		Psychosis (−)	5.7	15.6	27.6	38.8	50.2	61.1	71.0	77.7	82.2	86.7	93.2	<0.0001
		Psychosis (+)	0.5	2.5	5.9	16.3	30.5	45.4	56.4	66.6	78.1	88.5	93.9	
		Extra levodopa first	3.5	9.9	19.4	30.5	42.5	55.8	65.9	72.3	79.0	86.1	92.3	<0.0001
		Levodopa first	7.1	19.1	31.2	42.3	54.0	62.8	72.5	81.0	87.7	89.8	95.0	

Hoehn and Yahr stage 4	48.6% 11.7 (7.6) years	**Total**	**2.5**	**5.1**	**9.7**	**16.8**	**24.6**	**34.5**	**42.4**	**51.4**	**59.2**	**65.1**	**79.5**	
		<60.0 years old	1.2	2.5	2.8	6.1	8.5	15.2	21.2	30.4	39.7	48.4	68.7	<0.0001
		≥60.0 years old	2.9	7.8	16.4	28.0	42.6	58.0	70.8	82.1	90.2	90.2	100.0	
		Wearing-off (−)	6.8	12.6	21.8	31.4	41.6	52.8	58.3	63.0	70.8	74.6	84.9	<0.0001
		Wearing-off (+)	0.0	0.7	2.9	8.6	15.4	25.0	34.2	44.9	52.9	60.2	76.6	
		Extra levodopa first	1.6	3.3	5.8	13.2	21.0	31.0	38.8	47.1	55.2	62.4	78.0	0.001
		Levodopa first	4.1	8.0	16.0	22.8	30.5	40.3	48.3	58.2	65.3	69.6	81.9	

Pneumonia	9.0% 13.7 (7.4) years	**Total**	**0.2**	**0.3**	**1.0**	**1.9**	**3.3**	**5.2**	**7.6**	**11.9**	**13.3**	**16.4**	**24.8**	
		Male	0.2	0.4	1.3	2.5	5.1	6.4	11.1	16.0	19.3	19.7	26.2	0.003
		Female	0.0	0.1	0.6	1.1	1.7	2.8	4.3	8.1	10.3	12.8	20.0	
		<60.0 years old	0.0	0.0	0.2	0.3	0.7	1.3	2.7	4.1	6.3	7.3	14.2	<0.0001
		≥60.0 years old	0.2	0.5	1.5	3.3	6.2	8.6	15.2	30.4	39.6	49.6	74.8	
		Wearing-off (−)	0.2	0.5	2.8	5.1	8.5	10.3	13.7	18.3	24.9	24.9	34.0	<0.0001
		Wearing off (+)	0.0	0.1	0.7	1.3	2.5	3.7	6.4	10.5	13.2	14.7	21.0	
		Extra levodopa first	0.1	0.1	0.4	0.7	1.5	2.5	6.2	11.0	13.8	13.8	21.7	0.034
		Levodopa first	0.0	0.4	1.8	3.6	6.1	7.7	9.4	12.8	15.9	19.2	24.7	

Tube feeding	6.6% 16.1 (8.5) years	**Total**	**0.0**	**0.0**	**0.3**	**0.9**	**1.9**	**3.3**	**4.4**	**4.8**	**8.5**	**11.4**	**19.6**	
		<60.0 years old	0.0	0.0	0.0	0.0	0.2	0.8	1.0	2.0	2.0	4.0	13.5	<0.0001
		≥60.0 years old	0.0	0.0	0.5	1.8	3.8	6.5	9.8	20.4	29.4	45.7	—	
		Wearing-off (−)	0.0	0.0	1.0	1.9	3.4	4.9	6.0	8.2	13.7	17.4	29.0	0.027
		Wearing-off (+)	0.0	0.0	0.0	0.5	1.3	2.6	3.6	6.4	7.2	9.8	18.2	
		Extra levodopa first	0.0	0.0	0.1	0.1	1.0	1.6	2.7	6.0	7.0	9.2	19.6	0.048
		Levodopa first	0.0	0.0	0.5	2.0	2.9	5.6	6.5	9.5	10.1	11.2	20.2	

Death	10.8% 14.5 (8.0) years	**Total**	**0.0**	**0.0**	**0.3**	**1.6**	**2.3**	**4.0**	**6.2**	**8.7**	**11.0**	**14.3**	**21.1**	
		<60.0 years old	0.0	0.0	0.0	0.4	0.4	0.5	1.3	2.2	4.4	6.8	13.6	<0.0001
		≥60.0 years old	0.0	0.0	0.4	2.7	4.6	8.5	14.3	22.8	24.4	35.1	—	
		Pain (−)	0.0	0.0	0.3	1.2	2.2	4.3	6.9	10.5	13.1	16.9	22.8	0.039
		Pain (+)	0.0	0.0	0.0	2.0	2.3	3.1	4.4	5.1	6.6	8.7	14.1	
		Wearing-off (−)	0.0	0.0	0.7	3.5	5.1	7.0	10.1	14.2	14.2	18.7	39.0	0.003
		Wearing-off (+)	0.0	0.0	0.0	0.8	1.4	2.9	4.9	7.2	9.7	12.7	17.5	
		Psychosis (−)	0.0	0.0	0.3	1.3	2.0	3.0	4.4	6.6	7.5	10.8	15.8	0.041
		Psychosis (+)	0.0	0.0	0.0	1.6	2.6	4.8	7.7	10.5	13.6	16.8	21.5	

**Table 3:** Cox proportional-hazards model for clinical events in patients with Parkinson's disease.

	Sex (female)			Age at onset			Tremor onset			Daily dose of levodopa of first evaluation			Duration to the start of levodopa			Duration to the start of antiparkinsonian drugs except levodopa			Total LED at first evaluation			Duration of onset to pain			Duration of onset to wearing-off			Duration of onset to psychosis			Duration of onset to orthostatic hypotension		
Events	*p*	Hazard ratio	95% CI	*p*	Hazard ratio	95% CI	*p*	Hazard ratio	95% CI	*p*	Hazard ratio	95% CI	*p*	Hazard ratio	95% CI	*p*	Hazard ratio	95% CI	*p*	Hazard ratio	95% CI	*p*	Hazard ratio	95% CI	*p*	Hazard ratio	95% CI	*p*	Hazard ratio	95% CI	*p*	Hazard ratio	95% CI
Pain	**0.017**	**1.298**	**1.048–1.608**	0.059	1.012	1.000–1.024	0.426	1.089	0.883–1.342	0.090	0.999	0.999–1.000	0.817	0.995	0.954–1.038	**0.044**	**0.966**	**0.934–0.999**	0.007	1.001	1.000–1.001				**0.005**	**0.964**	**0.939–0.989**	**0.003**	**0.969**	**0.949–0.989**	**0.005**	**0.966**	**0.943–0.990**
Wearing-off	**<0.001**	**1.414**	**1.231–1.625**	**<0.001**	**0.968**	**0.960–0.975**	**0.001**	**0.785**	**0.683–0.902**	**0.033**	**0.999**	**0.999–1.000**	**<0.001**	**0.919**	**0.896–0.942**	0.195	0.987	0.967–1.007	**<0.001**	**1.001**	**1.000–1.001**	**<0.001**	**0.964**	**0.950–0.978**				**0.139**	**0.988**	**0.973–1.004**	**<0.001**	**0.946**	**0.931–0.962**
Camptocormia	0.154	1.222	0.986–1.017	0.863	1.001	0.986–1.017	0.278	0.861	0.658–1.128	0.319	1.000	0.999–1.000	0.640	0.988	0.938–1.000	0.122	0.964	0.921–1.010	0.728	1.000	0.999–1.001	**0.001**	**0.951**	**0.924–0.979**	0.190	0.976	0.941–1.012	0.375	0.986	0.954–1.018	0.736	1.007	0.968–1.047
Psychosis	0.725	0.967	0.804–1.164	**<0.001**	**1.035**	**1.023–1.047**	0.390	1.082	0.904–1.296	**0.037**	**1.001**	**1.000–1.001**	0.629	0.992	0.959–1.025	0.936	1.000	0.974–4.026	0.451	1.000	0.999–1.000	**<0.001**	**0.955**	**0.938–0.973**	**0.042**	**0.977**	**0.955–0.999**				**<0.001**	**0.941**	**0.921–0.962**
Orthostatic hypotension	**<0.001**	**0.540**	**0.382–0.763**	**0.002**	**1.033**	**1.012–1.054**	0.647	0.922	0.651–1.305	0.881	1.000	0.999–1.001	**0.029**	**0.930**	**0.872–0.993**	0.123	1.037	0.990–1.086	0.599	1.000	0.999–1.017	0.365	0.986	0.957–1.017	**0.034**	**0.957**	**0.918–0.997**	**<0.001**	**0.940**	**0.911–0.969**			
Modified Hoehn and Yahr stage 3	**0.005**	**1.211**	**1.059–1.386**	**<0.001**	**1.048**	**1.039–1.057**	**0.004**	**0.821**	**0.718–0.939**	0.211	1.000	1.000–1.001	0.087	0.977	0.952–1.003	0.273	1.011	0.991–1.032	0.655	1.000	1.000–1.000	**<0.001**	**0.960**	**0.946–0.974**	**<0.001**	**0.952**	**0.935–0.969**	**<0.001**	**0.955**	**0.940–0.970**	**<0.001**	**0.927**	**0.911–0.943**
Modified Hoehn and Yahr stage 4	**0.047**	**1.019**	**1.002–1.420**	**<0.001**	**1.071**	**1.058–1.083**	**0.004**	**0.776**	**0.654–0.921**	**0.001**	**1.001**	**1.000–1.002**	0.328	0.984	0.953–1.016	**0.035**	**1.027**	**1.002–1.052**	0.628	1.000	0.999–1.000	**<0.001**	**0.954**	**0.937–0.971**	**0.010**	**0.972**	**0.952–0.993**	**<0.001**	**0.934**	**0.918–0.952**	**0.002**	**0.968**	**0.948–0.988**
Pneumonia	**0.001**	**0.510**	**0.342–0.760**	**<0.001**	**1.122**	**1.091–1.154**	0.355	0.828	0.555–1.235	0.996	1.000	0.999–1.001	0.662	0.984	0.913–1.059	0.970	1.001	0.949–1.056	0.572	1.000	0.999–1.001	0.494	0.987	0.950–1.025	0.199	0.971	0.928–1.016	0.294	0.982	0.950–1.016	**0.001**	**0.982**	**0.893–0.972**
Tube feeding	0.914	1.027	0.630–1.675	**<0.001**	**1.136**	**1.096–1.177**	0.175	0.709	0.431–1.165	0.858	1.000	0.999–1.002	0.862	1.007	0.928–1.093	0.468	1.021	0.965–1.093	0.614	1.000	0.999–1.002	0.344	0.979	0.937–1.023	0.407	0.979	0.930–1.030	0.446	0.986	0.950–1.023	**<0.001**	**0.913**	**0.870–0.957**
Death	0.715	0.921	0.592–1.133	**<0.001**	**1.096**	**1.063–1.130**	0.418	0.831	0.531–1.130	0.670	1.000	0.998–1.001	0.847	1.008	0.928–1.095	0.441	1.022	0.966–1.082	0.611	1.000	0.999–1.001	**0.001**	**0.935**	**0.901–1.019**	0.217	0.969	0.921–1.019	0.080	0.968	0.934–1.004	**<0.001**	**0.893**	**0.854–0.933**

**Table 4:** Subgroup analysis of Cox proportional-hazards model divided by gender for clinical events in patients with Parkinson's disease.

Subgroup analysis	Events	Age at onset			Tremor onset			Daily dose of levodopa at first evaluation			Duration until the start of levodopa			Duration until the start of antiparkinsonian drugs except levodopa			Total LED at first evaluation			Duration of onset to pain			Duration until onset of wearing-off			Duration until onset of psychosis			Duration until onset of orthostatic hypotension		
		*p*	Hazard ratio	95% CI	*p*	Hazard ratio	95% CI	*p*	Hazard ratio	95% CI	*p*	Hazard ratio	95% CI	*p*	Hazard ratio	95% CI	*p*	Hazard ratio	95% CI	*p*	Hazard ratio	95% CI	*p*	Hazard ratio	95% CI	*p*	Hazard ratio	95% CI	*p*	Hazard ratio	95% CI
Male	Pain	0.185	1.014	0.993–1.034	0.119	1.312	0.932–1.847	0.054	0.999	0.998–1.000	0.294	0.964	0.900–1.032	0.882	1.004	0.951–1.060	**0.005**	**1.001**	**1.000–1.002**				**0.024**	**0.951**	**0.911–0.993**	0.088	0.971	0.939–1.004	**0.020**	**0.950**	**0.911–0.992**
	Wearing-off	**<0.001**	0.952	0.940–0.965	**0.012**	**0.755**	**0.606–0.941**	0.247	1.000	0.999–1.000	**<0.001**	**0.921**	**0.886–0.957**	0.242	0.981	0.950–1.013	**0.016**	**1.001**	**1.000–1.001**	**<0.001**	0.951	0.928–0.975	**0.013**	**0.972**	**0.950–0.994**	**0.013**	**0.972**	**0.950–0.994**	**<0.001**	**0.950**	**0.925–0.975**
	Camptocormia	0.558	1.008	0.982–1.035	0.691	0.913	0.582–1.431	0.379	0.999	0.998–1.001	0.232	1.054	0.967–1.150	0.075	0.925	0.849–1.008	0.857	1.000	0.999–1.001	0.050	0.949	0.875–0.994	**0.032**	**0.933**	**0.875–0.994**	0.403	1.020	0.973–1.070	0.517	1.023	0.955–1.095
	Psychosis	**<0.001**	**1.041**	**0.023–1.059**	**0.032**	**1.354**	**1.026–1.785**	0.076	1.001	1.000–1.002	0.595	1.015	0.961–1.071	0.791	1.007	0.959–1.056	0.304	1.000	0.999–1.000	**0.046**	**0.969**	**0.939–0.999**	**<0.001**	**0.934**	**0.902–0.968**				**0.008**	**0.951**	**0.917–0.987**
	Orthostatic hypotension	**0.012**	**1.037**	**1.008–1.067**	0.295	0.774	0.479–1.250	0.624	1.000	0.998–1.001	0.165	0.942	0.865–1.025	0.297	1.036	0.969–1.109	0.571	1.000	0.999–1.002	0.082	0.961	0.919–1.005	**0.045**	**0.939**	**0.884–0.999**	0.057	0.958	0.916–1.001			
	Modified Hoehn and Yahr stage 3	**<0.001**	**1.042**	**1.028–1.055**	**0.006**	**0.745**	**0.602–0.921**	0.150	1.001	1.000–1.001	0.515	0.986	0.945–1.029	0.238	1.023	0.985–1.029	0.287	1.000	0.999–1.000	**<0.001**	**0.947**	**0.923–0.972**	**<0.001**	**0.948**	**0.923–0.975**	**<0.001**	**0.951**	**0.929–0.974**	**<0.001**	**0.914**	**0.889–0.940**
	Modified Hoehn and Yahr stage 4	**<0.001**	**1.059**	**1.040–1.078**	**0.002**	**0.644**	**0.489–0.846**	**0.017**	**1.001**	**1.000–1.002**	0.970	0.999	0.950–1.051	0.190	1.029	0.986–1.075	0.366	1.000	0.999–1.000	**0.001**	**0.952**	**0.923–0.981**	**0.027**	**0.961**	**0.928–0.996**	**<0.001**	**0.931**	**0.906–0.957**	0.065	0.970	0.939–1.002
	Pneumonia	**<0.001**	**1.100**	**1.060–1.142**	0.406	0.791	0.456–1.374	0.556	0.999	0.997–1.001	0.866	0.991	0.892–1.101	0.093	0.920	0.834–1.014	0.573	1.000	0.999–1.002	0.796	1.008	0.950–1.070	0.570	0.981	0.919–1.048	0.173	0.964	0.915–1.016	**<0.001**	**0.887**	**0.838–0.940**
	Tube feeding	**0.016**	**1.069**	**1.013–1.128**	**0.018**	**0.342**	**0.141–0.831**	0.463	1.001	0.998–1.004	0.775	0.978	0.836–1.143	0.636	0.969	0.853–1.102	0.321	0.999	0.996–1.001	0.512	0.973	0.896–1.056	0.488	1.033	0.942–1.133	0.198	0.959	0.900–1.022	**<0.001**	**0.844**	**0.783–0.910**
	Death	**0.001**	**1.084**	**1.034–1.138**	0.394	0.737	0.365–1.488	0.962	1.000	0.998–1.002	0.704	1.025	0.903–1.164	0.862	0.991	0.898–1.094	0.650	1.000	0.999–1.002	0.231	0.964	0.907–1.024	0.172	0.944	0.870–1.025	0.161	0.962	0.912–1.015	**0.001**	**0.893**	**0.836–0.954**

Female	Pain	0.135	1.012	0.996–1.028	0.899	0.983	0.753–1.284	0.402	1.000	0.999–1.000	0.357	1.036	0.961–1.117	0.196	0.944	0.865–1.030	0.126	1.001	1.000–1.001				0.017	0.962	0.932–0.993	**0.040**	**0.972**	**0.946–0.999**	0.032	0.966	0.936–0.997
	Wearing-off	**<0.001**	**0.975**	**0.965–0.985**	**0.027**	**0.816**	**0.682–0.977**	0.165	1.000	0.999–1.000	**<0.001**	**0.906**	**0.861–0.954**	0.636	1.014	0.956–1.076	**<0.001**	**1.001**	**1.001–1.001**	**0.001**	**0.970**	**0.953–0.988**				0.699	0.996	0.974–1.018	**<0.001**	**0.939**	**0.918–0.960**
	Camptocormia	0.997	1.000	0.981–1.019	0.363	0.854	0.608–1.200	0.774	1.000	0.999–1.001	0.822	1.011	0.921–1.109	**0.028**	**0.869**	**0.768–0.985**	0.412	1.000	0.999–1.001	**0.005**	**0.951**	**0.918–0.985**	0.963	0.999	0.955–1.045	0.102	0.965	0.924–1.007	0.722	1.009	0.960–1.060
	Psychosis	**0.001**	**1.027**	**1.012–1.043**	0.424	0.907	0.714–1.152	0.392	1.000	1.000–1.001	0.112	0.948	0.887–1.013	0.649	1.017	0.946–1.092	0.753	1.000	0.999–1.001	**<0.001**	**0.951**	**0.929–0.973**	0.412	1.013	0.982–1.045				**<0.001**	**0.918**	**0.890–0.947**
	Orthostatic hypotension	**0.047**	**1.032**	**1.000–1.064**	0.906	1.032	0.612–1.738	0.749	1.000	0.999–1.002	0.754	0.974	0.825–1.149	0.456	0.929	0.765–1.128	0.862	1.000	0.999–1.001	0.674	1.009	0.967–1.054	0.461	0.980	0.927–1.035	**0.002**	**0.930**	**0.888–0.973**			
	Modified Hoehn and Yahr stage 3	**<0.001**	**1.051**	**1.039–1.063**	0.174	0.884	0.740–1.056	0.807	1.000	0.999–1.001	**0.024**	**0.944**	**0.898–0.992**	0.210	0.944	0.981–1.091	0.641	1.000	1.000–1.001	**0.001**	**0.968**	**0.951–0.986**	**0.001**	**0.959**	**0.935–0.982**	**<0.001**	**0.955**	**0.933–0.979**	**<0.001**	**0.930**	**0.906–0.955**
	Modified Hoehn and Yahr stage 4	**<0.001**	**1.080**	**1.063–1.097**	0.517	0.929	0.742–1.162	**0.014**	**1.001**	**1.000–1.002**	**0.037**	**0.932**	**0.872–0.996**	0.276	1.040	0.969–1.115	0.820	1.000	0.999–1.001	**<0.001**	**0.955**	**0.934–0.976**	0.435	0.989	0.962–1.017	**<0.001**	**0.933**	**0.910–0.958**	**0.008**	**0.963**	**0.936–0.990**
	Pneumonia	**<0.001**	**1.141**	**1.092–1.193**	0.731	0.899	0.489–1.652	0.516	1.001	0.999–1.003	0.333	0.915	0.764–1.095	0.171	1.131	0.948–1.349	0.774	1.000	0.999–1.002	0.367	0.976	0.926–1.029	0.318	0.966	0.901–1.034	0.977	1.001	0.942–1.063	0.281	0.960	0.893–1.034
	Tube feeding	**<0.001**	**1.173**	**1.116–1.232**	0.545	1.223	0.637–2.347	0.958	1.000	0.998–1.002	0.678	0.956	0.774–1.182	0.444	1.088	0.876–1.353	0.205	1.001	0.999–1.003	0.584	0.985	0.932–1.041	0.129	0.944	0.877–1.017	0.874	0.995	0.937–1.057	0.199	0.953	0.885–1.026
	Death	**<0.001**	**1.111**	**1.065–1.158**	0.707	0.891	0.488–1.627	0.199	0.999	0.996–1.001	0.061	0.832	0.687–1.009	0.130	1.153	0.959–1.386	0.269	1.001	0.999–1.002	0.003	0.922	0.875–0.973	0.591	0.979	0.906–1.058	0.625	0.984	0.920–1.051	**<0.001**	**0.847**	**0.790–0.908**

**Table 5:** Subgroup analysis of Cox proportional-hazards models divided by onset age for clinical events in patients with Parkinson's disease.

Subgroup analysis	Events	Sex (female)			Tremor onset			Daily dose of levodopa at first evaluation			Duration until the start of levodopa			Duration until the start of antiparkinsonian drugs except levodopa			Total LED at first evaluation			Duration of onset to pain			Duration until onset of wearing-off			Duration until onset of psychosis			Duration until onset of orthostatic hypotension		
		*p*	Hazard ratio	95% CI	*p*	Hazard ratio	95% CI	*p*	Hazard ratio	95% CI	*p*	Hazard ratio	95% CI	*p*	Hazard ratio	95% CI	*p*	Hazard ratio	95% CI	*p*	Hazard ratio	95% CI	*p*	Hazard ratio	95% CI	*p*	Hazard ratio	95% CI	*p*	Hazard ratio	95% CI
Age at onset under 60 years	Pain	**0.017**	**1.426**	**1.064–1.910**	0.388	0.878	0.655–1.179	0.238	1.000	0.999–1.000	0.859	1.005	0.955–1.000	**0.048**	**0.960**	**0.922–1.000**	**0.015**	**1.001**	**1.000–1.001**				0.064	0.972	0.943–1.002	**0.010**	**0.970**	**0.948–0.993**	0.006	0.963	0.937–0.989
	Wearing-off	**0.001**	**1.368**	**1.139–1.644**	**<0.001**	**0.663**	**0.551–0.799**	**0.010**	**0.999**	**00999–1.000**	**<0.001**	**0.930**	**0.904–0.958**	0.493	0.992	0.969–1.015	**<0.001**	**1.001**	**1.001–1.001**	**<0.001**	**0.966**	**0.950–0.982**				0.703	0.997	0.979–1.014	**<0.001**	**0.963**	**0.945–0.980**
	Camptocormia	0.902	0.977	0.676–1.412	0.730	0.937	0.649–1.355	0.139	1.001	1.000–1.002	0.775	1.009	0.950–1.071	0.495	0.982	0.933–1.034	0.076	0.999	0.998–1.000	**0.026**	**0.961**	**0.929–0.995**	0.325	0.979	0.937–1.022	0.315	0.981	0.946–1.018	0.620	0.989	0.945–1.034
	Psychosis	0.387	1.125	0.862–1.468	0.319	1.141	0.8880–1.480	**0.019**	**1.001**	**1.000–1.002**	0.053	0.962	0.926–1.000	0.932	0.999	0.969–1.029	0.318	1.000	0.999–1.000	**<0.001**	**0.947**	**0.926–0.968**	0.283	1.015	0.988–1.042				**<0.001**	**0.952**	**0.929–0.976**
	Orthostatic hypotension	0.061	0.636	0.397–1.021	0.841	1.050	0.649–1.700	0.642	1.000	0.998–1.001	**0.046**	**0.926**	**0.858–0.998**	0.256	1.032	0.978–1.089	0.335	1.000	0.999–1.001	0.200	0.977	0.944–1.012	0.170	0.967	0.922–1.014	**0.001**	**0.943**	**0.911–0.975**			
	Modified Hoehn and Yahr stage 3	0.108	1.178	0.965–1.437	0.280	0.896	0.734–1.094	**0.026**	**1.001**	**1.000–1.001**	0.107	0.974	0.943–1.006	0.551	1.008	0.983–1.033	0.126	1.000	0.999–1.000	**<0.001**	**0.958**	**0.941–0.975**	**0.021**	**0.977**	**0.957–0.996**	**<0.001**	**0.948**	**0.932–0.965**	**<0.001**	**0.933**	**0.914–0.952**
	Modified Hoehn and Yahr stage 4	0.229	1.177	0.902–1.535	0.203	0.842	0.646–1.097	**<0.001**	**1.002**	**1.001–1.001**	0.318	0.980	0.942–1.020	0.306	1.016	0.985–1.048	0.064	0.999	0.999–1.000	**<0.001**	**0.955**	**0.934–0.975**	0.872	0.998	0.975–1.022	**<0.001**	**0.926**	**0.905–0.946**	**0.003**	**0.963**	**0.940–0.987**
	Pneumonia	0.164	0.638	0.338–1.202	0.892	0.956	0.501–1.827	0.063	1.002	1.000–1.003	0.659	0.979	0.893–1.074	0.649	1.015	0.920–1.011	0.203	0.999	0.998–1.001	0.131	0.964	0.920–1.011	0.716	1.010	0.956–1.068	0.232	0.976	0.937–1.016	**0.001**	**0.914**	**0.869–0.962**
	Tube feeding	0.688	1.172	0.540–2.546	0.396	0.706	0.316–1.577	**0.021**	**1.002**	**1.000–1.004**	0.436	1.039	0.944–1.143	0.857	1.006	0.942–1.074	0.127	0.999	0.997–1.000	0.332	0.973	0.921–1.028	0.361	0.973	0.921–1.028	0.265	0.976	0.936–1.018	**<0.001**	**0.888**	**0.841–0.938**
	Death	0.459	0.770	0.384–1.541	0.701	0.867	0.418–1.797	0.964	1.000	0.998–1.002	0.915	0.995	0.906–1.092	0.304	1.034	0.970–1.103	0.877	1.0000	0.999–1.002	**0.003**	**0.93**	**0.887–0.975**	0.249	1.035	0.976–1.097	0.048	0.962	0.925–1.000	**<0.001**	**0.897**	**0.853–0.944**

Age at onset over 60 years	Pain	0.249	1.205	0.878–1.656	**0.026**	**1.414**	**1.042–1.919**	0.404	0.999	0.998–1.001	0.224	0.953	0.883–1.030	0.418	0.973	0.911–1.039	0.362	1.000	1.000–1.001				0.092	0.959	0.914–1.007	**0.032**	**0.953**	**0.913–0.996**	0.066	0.953	0.906–1.003
	Wearing-off	**<0.001**	**1.509**	**1.213–1.877**	0.189	0.870	0.706–1.071	**0.010**	**0.999**	**0.998–1.000**	**0.002**	**0.924**	**0.879–0.972**	**0.038**	**0.954**	**0.913–0.997**	**<0.001**	**1.001**	**1.001–1.002**	0.077	0.974	0.945–1.003				0.279	0.982	0.949–1.015	**0.001**	**0.940**	**0.906–0.975**
	Camptocormia	0.042	1.556	1.016–2.381	0.190	0.767	0.515–1.141	**0.001**	**0.997**	**0.996–0.999**	**0.047**	**0.894**	**0.801–0.999**	**0.032**	**0.897**	**0.813–0.991**	0.148	1.001	1.000–1.002	**0.001**	**0.917**	**0.870–0.967**	0.396	0.971	0.907–1.040	0.817	0.992	0.926–1.063	0.154	1.059	0.979–1.146
	Orthostatic hypotension	**0.006**	**0.492**	**0.297–0.814**	0.568	0.863	0.520–1.431	0.205	1.001	0.999–1.003	0.099	0.898	0.790–1.020	0.794	1.013	0.919–1.118	0.137	0.999	0.997–1.000	0.957	0.998	0.936–1.065	0.154	0.947	0.879–1.021	**0.005**	**0.912**	**0.856–0.973**			
	Psychosis	0.434	0.903	0.699–1.166	0.966	1.006	0.780–1.297	0.081	1.001	1.000–1.002	0.905	1.004	0.944–1.067	0.617	0.987	0.937–1.039	0.262	1.000	0.999–1.000	**0.004**	**0.953**	**0.921–0.985**	**0.002**	**0.938**	**0.901–0.977**				**<0.001**	**0.878**	**0.840–0.917**
	Modified Hoehn and Yahr stage 3	**0.001**	**1.375**	**1.143–1.654**	**0.010**	**0.787**	**0.656–0.944**	0.064	1.001	1.000–1.001	**0.001**	**0.920**	**0.878–0.964**	0.757	0.994	0.957–1.032	0.192	1.000	0.999–1.000	**0.002**	**0.955**	**0.929–0.983**	**0.001**	**0.948**	**0.917–0.983**	**0.012**	**0.961**	**0.932–0.991**	**<0.001**	**0.875**	**0.845–0.905**
	Modified Hoehn and Yahr stage 4	0.082	1.228	0.975–1.547	**0.024**	**0.770**	**0.613–0.967**	0.072	1.001	1.000–1.002	0.056	0.946	0.894–1.001	0.267	1.025	0.982–1.070	0.416	1.000	0.999–1.000	**<0.001**	**0.936**	**0.909–0.963**	**0.015**	**0.953**	**0.917–0.991**	**<0.001**	**0.943**	**0.913–0.974**	**<0.001**	**0.942**	**0.910–0.974**
	Pneumonia	**0.011**	**0.513**	**0.307–0.858**	0.563	0.859	0.515–1.436	0.864	1.000	0.998–1.002	0.065	0.893	0.792–1.007	0.971	0.998	0.912–1.093	0.840	1.000	0.999–1.002	0.886	1.005	0.944–1.069	0.373	0.967	0.900–1.040	0.319	0.968	0.907–1.032	**0.001**	**0.883**	**0.824–0.947**
	Tube feeding	0.801	1.086	0.571–2.069	0.610	0.847	0.449–1.601	0.465	0.999	0.997–1.002	**0.048**	**0.844**	**0.714–0.998**	0.471	1.041	0.934–1.159	0.513	1.001	0.999–1.002	0.510	0.976	0.907–1.050	0.154	0.935	0.852–1.026	0.567	0.976	0.899–1.060	**0.002**	**0.876**	**0.804–0.953**
	Death	0.765	1.094	0.609–1.964	0.579	0.850	0.477–1.512	0.659	1.001	0.998–1.003	0.575	0.955	0.814–1.121	0.883	0.991	0.879–1.117	0.467	0.999	0.998–1.001	**0.026**	**0.931**	**0.874–0.952**	**0.003**	**0.866**	**0.788–0.952**	0.791	0.989	0.914–1.071	**<0.001**	**0.821**	**0.761–0.887**

## Data Availability

The data used to support the findings of this study are available from the corresponding author upon request.

## References

[1] Ben-Shlomo Y., Marmot M. G. (1995). Survival and cause of death in a cohort of patients with parkinsonism: possible clues to aetiology?. *Journal of Neurology, Neurosurgery & Psychiatry*.

[2] Diem-Zangerl A., Seppi K., Wenning G. K. (2009). Mortality in Parkinson’s disease: a 20-year follow-up study. *Movement Disorders*.

[3] Hely M. A., Morris J. G. L., Reid W. G. J., Trafficante R. (2005). Sydney multicenter study of Parkinson’s disease: non-L-dopa-responsive problems dominate at 15 years. *Movement Disorders*.

[4] Katzenschlager R., Head J., Schrag A. (2008). Fourteen-year final report of the randomized PDRG-UK trial comparing three initial treatments in PD. *Neurology*.

[5] Hauser R. A., Lew M. F., Hurtig H. I. (2009). Long-term outcome of early versus delayed rasagiline treatment in early Parkinson’s disease. *Movement Disorders*.

[6] Picillo M., Palladino R., Moccia M. (2016). Gender and non motor fluctuations in Parkinson’s disease: a prospective study. *Parkinsonism & Related Disorders*.

[7] Erro R., Picillo M., Vitale C. (2016). The non-motor side of the honeymoon period of Parkinson’s disease and its relationship with quality of life: a 4-year longitudinal study. *European Journal of Neurology*.

[8] Prange S., Danaila T., Laurencin C. (2019). Age and time course of long-term motor and nonmotor complications in Parkinson disease. *Neurology*.

[9] Yoritaka A., Shimo Y., Takanashi M. (2013). Motor and non-motor symptoms of 1453 patients with Parkinson’s disease: prevalence and risks. *Parkinsonism & Related Disorders*.

[10] Yoritaka A., Shimo Y., Takanashi M. (2014). Motor and non-motor symptoms of 1453 patients with Parkinson’s disease: any relationship with the cumulative doses of anti-parkinsonian medications?. *Journal of Neurological Disorders & Stroke*.

[11] Hughes A. J., Daniel S. E., Kilford L., Lees A. J. (1992). Accuracy of clinical diagnosis of idiopathic Parkinson’s disease: a clinico-pathological study of 100 cases. *Journal of Neurology, Neurosurgery & Psychiatry*.

[12] Chaudhuri K. R., Martinez-Martin P., Brown R. G. (2007). The metric properties of a novel non-motor symptoms scale for Parkinson’s disease: results from an international pilot study. *Movement Disorders*.

[13] Visser M., Verbaan D., van Rooden S. M., Stiggelbout A. M., Marinus J., van Hilten J. J. (2007). Assessment of psychiatric complications in Parkinson’s disease: the SCOPA-PC. *Movement Disorders*.

[14] Marinus J., Visser M., Verwey N. A. (2003). Assessment of cognition in Parkinson’s disease. *Neurology*.

[15] Tomlinson C. L., Stowe R., Patel S., Rick C., Gray R., Clarke C. E. (2010). Systematic review of levodopa dose equivalency reporting in Parkinson’s disease. *Movement Disorders*.

[16] Williams D. R., de Silva R., Paviour D. C. (2005). Characteristics of two distinct clinical phenotypes in pathologically proven progressive supranuclear palsy: richardson’s syndrome and PSP-parkinsonism. *Brain*.

[17] Ministry of Health, Labour and Welfare (2013). Himan te donnajyoutai?. https://web.archive.org/web/20130628232937/http://www.mhlw.go.jp/topics/bukyoku/kenkou/seikatu/himan/about.html.

[18] Sato K., Hatano T., Yamashiro K. (2006). Prognosis of Parkinson’s disease: time to stage III, IV, V, and to motor fluctuations. *Movement Disorders*.

[19] Haaxma C. A., Bloem B. R., Borm G. F. (2007). Gender differences in Parkinson’s disease. *Journal of Neurology, Neurosurgery & Psychiatry*.

[20] Stocchi F., Antonini A., Barone P. (2014). Early DEtection of wEaring off in Parkinson disease: the DEEP study. *Parkinsonism & Related Disorders*.

[21] Schrag A., Quinn N. (2000). Dyskinesias and motor fluctuations in Parkinson’s disease. *Brain*.

[22] Kumagai T., Nagayama H., Ota T., Nishiyama Y., Mishina M., Ueda M. (2014). Sex differences in the pharmacokinetics of levodopa in elderly patients with Parkinson disease. *Clinical Neuropharmacology*.

[23] Olanow C. W., Kieburtz K., Rascol O. (2013). Factors predictive of the development of levodopa-induced dyskinesia and wearing-off in Parkinson’s disease. *Movement Disorders*.

[24] Cilia R., Akpalu A., Sarfo F. S. (2014). The modern pre-levodopa era of Parkinson’s disease: insights into motor complications from sub-Saharan Africa. *Brain*.

[25] Kempster P. A., O’Sullivan S. S., Holton J. L., Revesz T., Lees A. J. (2010). Relationships between age and late progression of Parkinson’s disease: a clinico-pathological study. *Brain*.

[26] Baker W. L., Silver D., White C. M. (2009). Dopamine agonists in the treatment of early Parkinson’s disease: a meta-analysis. *Parkinsonism & Related Disorders*.

[27] De Pablo-Fernandez E., Tur C., Revesz T., Lees A. J., Holton J. L., Warner T. T. (2017). Association of autonomic dysfunction with disease progression and survival in Parkinson disease. *JAMA Neurology*.

[28] Fereshtehnejad S.-M., Romenets S. R., Anang J. B. M., Latreille V., Gagnon J.-F., Postuma R. B. (2015). New clinical subtypes of Parkinson disease and their longitudinal progression. *JAMA Neurology*.

[29] Chung S. J., Bae Y. J., Jun S. Y. (2019). Dysautonomia is associated with structural and functional alterations in Parkinson disease. *Neurology*.

[30] Pinter B., Diem-Zangerl A., Wenning G. K. (2015). Mortality in Parkinson’s disease: a 38-year follow-up study. *Movement Disorders*.

[31] Ba F., Obaid M., Wieler M., Camicioli R., Martin W. R. W. (2016). Parkinson disease: the relationship between non-motor symptoms and motor phenotype. *Canadian Journal of Neurological Sciences/Journal Canadien des Sciences Neurologiques*.

[32] Georgiev D., Hamberg K., Hariz M., Forsgren L., Hariz G. M. (2017). Gender differences in Parkinson’s disease: a clinical perspective. *Acta Neurologica Scandinavica*.

